# Building a committed workforce: the synergistic effects of coaching leadership, organizational self-esteem, and learning goal orientation

**DOI:** 10.3389/fpsyg.2024.1423540

**Published:** 2024-07-09

**Authors:** Leilei Tang, Mengjuan Shi, Yu Liu, Yizhi Liu, Bingcheng Yang

**Affiliations:** ^1^School of Marxism, Guizhou University, Guiyang, China; ^2^School of Management, Guizhou University, Guiyang, China; ^3^School of Foreign Languages, Wuhan Institute of Technology, Guiyang, China; ^4^School of Business Administration, Guizhou University of Finance and Economics, Guiyang, China

**Keywords:** coaching leadership, employee engagement, organizational self-esteem, learning goal orientation, synergistic effects

## Abstract

In today’s volatile, uncertain, complex, and ambiguous (VUCA) work environments, mitigating employee burnout and turnover has become a critical concern. The enhancement of employee engagement stands out as a pivotal focus in corporate human resource management. Coaching leadership focuses on the encouragement and inspiration of employees, which can effectively stimulate the internal potential of employees, enhance work ability and enhance engagement. However, previous research on the relationship between coaching leadership style and employee engagement are limited, thus obscures the essential function in enterprise development and core competitiveness. The research collected 402 valid responses from MBA and EMBA students at the School of Business, and examines the effect of coaching leadership on employee engagement. Results indicate that coaching leadership significantly enhances multiple facets of employee engagement, including vigor, devotion, and absorption. Crucially, organizational self-esteem emerges as a mediating factor, while learning goal orientation strengthens the positive effects of coaching leadership. This research sheds light on the nuanced dynamics of effective leadership in contemporary workplaces, also it underscores the need for more nuanced, industry-specific analyses and broader exploration of moderating variables. Ultimately, the insights garnered hold profound implications for leadership training, human resource strategies, and performance metrics, emphasizing a more integrative and holistic approach to leadership and employee development in vocational contexts.

## 1 Introduction

In the swiftly evolving VUCA (Volatility, Uncertainty, Complexity, and Ambiguity) landscape, contemporary organizations are undergoing profound transformations in their shapes and frameworks (Borde et al., 2024). These transformations necessitate a novel breed of leadership—one that is flexible, adaptable, and adept at navigating an ever-more unstable external milieu (Hensellek et al., 2023). Conventional leadership paradigms, while formerly efficacious, now prove inadequate in addressing the complexities of the present-day challenges (Pizzolitto et al., 2023; Borde et al., 2024). The evolving terrain is additionally nuanced by the emergent values and expectations of the new generation of employees. This cohort, notably those born post-1995, exhibits a robust inclination toward self-realization, autonomy, and decision-making authority. They place unprecedented emphasis on the leadership styles of their superiors, which adds complexity to the evolving organizational landscape. The coaching leadership style is considered to be a promising leadership development practice (Ely et al., 2010), with more emphasis on guidance, inspiration and positive support, and has become a widely used leadership development intervention (Ladegard and Gjerde, 2014; Passarelli et al., 2023). Thus, coaching leadership has garnered notable attention in both academic discourse and management practice (Aghababaei, 2023). Characterized by its emphasis on encouragement, inspiration, and effective communication, coaching leadership emerges as a favored approach to engaging and empowering this new generation of employees (Ren et al., 2018).

While numerous studies have examined the influence of coaching leadership on various employee outcomes such as innovation, organizational citizenship, job satisfaction, and performance (Baird et al., 2020; Wee et al., 2020; Aghababaei, 2023), there remains a gap in understanding how this leadership style directly affects employee engagement—a crucial driver of organizational competitiveness (Schaufeli and Salanova, 2011; Kuan and Bakar, 2023), thus obscures the essential function of coaching leadership style in enterprise development and core competitiveness. Gallup’s expansive analysis across multiple industries corroborates the importance of employee engagement, associating higher engagement levels with a 13% increase in retention, a 5% boost in productivity, a 52% rise in customer satisfaction, and a notable 44% growth in profitability (Harter et al., 2002). In an environment marked by internal competition and evolving career structures such as boundaryless careers, employee engagement is becoming increasingly elusive (Anitha, 2014). Thus, the uncertainty of employee engagement combined with the high level of modern turnover rate poses a major challenge to modern enterprise management (Climek et al., 2024).

To tackle this challenge effectively, it is imperative to delve deeply into the effects of coaching leadership on employee engagement. This paper utilizes Social Exchange Theory as a foundational framework to thoroughly examine the synergistic relationship between coaching leadership and employee engagement. We clame that coaching leadership cultivates a culture of reciprocity, which enhances employees’ sense of organizational self-esteem and subsequently boosts their engagement levels. Notably, we acknowledge the diversity among employees, recognizing that they possess unique traits, needs, and values that profoundly shape their interactions with different leadership styles. Our research endeavors to elucidate the mechanisms underlying coaching leadership’s synergistic impact on employee engagement. Specifically, we introduce organizational self-esteem as a mediating variable and examine the role of learning goal orientation as a moderating factor in this relationship. In doing so, this study contributes to both the theoretical understanding and the practical application of coaching leadership, filling an existing research gap and offering actionable insights for human resource management practices.

The research extends the theoretical framework regarding leadership efficacy, and also provides insights for addressing leadership challenges in the VUCA environment. By revealing the synergistic effects of coaching leadership on employee engagement, and also contributes to revealing the process and conditions conducive to the effective implementation of coaching leadership behaviors. This holds significant practical significance for improving leadership styles within enterprises and alleviating employee burnout. Simultaneously, it provides theoretical insights that can inform management practices in navigating complex and dynamic environments.

## 2 Research hypotheses and model construction

### 2.1 Research hypotheses

#### 2.1.1 The impact of coaching leadership on employee engagement

Drawing from Social Exchange Theory, coaching leadership serves as a catalyst for creating reciprocal interactions between leaders and employees (Anitha, 2014). When leaders exhibit coaching behaviors, employees perceive them as supportive and approachable (Bedarkar and Pandita, 2014). This positive perception fosters emotional connections and initiates a cycle of mutual responsibilities that manifest through favorable work attitudes and behaviors (Kim and Kuo, 2015). Coaching leadership stands out as a pivotal element in achieving both individual and organizational success (Yukl and Mahsud, 2010). Numerous studies have revealed its beneficial impact on various employee outcomes, including psychological capital, work engagement, and happiness (Kelloway et al., 2013; Zbierowski and Góra, 2014; Alok, 2017), as well as organizational commitment (Al-Nasser, 2016) and role-based performance (Kim, 2014).

This leadership style places a strong emphasis on supporting employees by establishing open channels of communication and providing guidance and inspiration. Consequently, it serves multiple functions. Coaching leadership offers subordinates essential resources and informational support (Eldor and Vigoda-Gadot, 2017; Mäkelä et al., 2024). By valuing employee needs and promoting growth opportunities, coaching leadership enhances employees’ sense of control over organizational processes, thereby boosting their engagement levels (Chughtai and Buckley, 2011). Techniques such as encouragement, effective communication, and inspiration are employed to unlock employees’ latent potential (Eldor and Vigoda-Gadot, 2017). Through problem-solving assistance, constructive feedback, and soliciting employee input, coaching leadership ensures a mutually beneficial interaction, contributing to improved work capabilities and sustained vigor (Elloy, 2005). Furthermore, coaching leadership helps employees grasp the broader organizational goals, clarifies their roles (Yuan et al., 2019; Borde et al., 2024), and reinforces their sense of purpose. This heightened understanding ignites enthusiasm and dedication—critical elements that translate into higher engagement levels (Ferris et al., 2009). In addition, coaching leadership has been demonstrated to significantly enhance job satisfaction (Wee et al., 2020) and stimulate innovative behavior (Wang, 2013), among other positive outcomes. In summary, coaching leadership not only rejuvenates the workforce but also fosters a profound sense of responsibility and dedication, ultimately resulting in heightened levels of employee engagement.

Employee engagement, as defined by Schaufeli and Bakker, represents a deep and positive connection to one’s work that goes beyond mere involvement (Schaufeli et al., 2002). It is characterized by vigor, dedication, and absorption, wherein vigor signifies heightened energy levels, mental resilience, a readiness to invest effort, and the ability to persevere in challenging situations (Gruman and Saks, 2011). Dedication encompasses a sense of significance, enthusiasm, inspiration, pride, and an ongoing desire to confront challenges (Little and Little, 2006). It goes beyond basic involvement. Absorption, on the other hand, signifies complete concentration and deep immersion in one’s work, resulting in a distorted perception of time and difficulty in disengaging from tasks, closely resembling the concept of ‘flow’ (Sun and Bunchapattanasakda, 2019). Together, these elements form the foundation of engagement, a critical driver of employee well-being and performance.

Based on this understanding, we propose the following hypotheses:

*H1*: Coaching leadership has a significant positive impact on overall employee engagement.

*H1a*: Coaching leadership positively influences employee vigor.

*H1b*: Coaching leadership enhances employee dedication.

*H1c*: Coaching leadership contributes to increased employee Absorption.

The Impact of Coaching Leadership on Organizational Self-esteem.

Organizational self-esteem refers to an employee’s evaluation of their own value and role within the organizational context. Often, leaders are perceived as organizational proxies, embodying the values and standards of the workplace (Eisenberger et al., 2010). High-quality coaching leadership emphasizes effective communication through methods like guidance, inspiration, and encouragement. This fosters an enhanced perception of one’s role within the organization (Yuan et al., 2019). Furthermore, coaching leadership cultivates employees’ sense of competence and environmental mastery, thereby fostering organizational identification and enhancing organizational self-esteem. Moreover, such leaders instill confidence in their employees by clarifying objectives, reinforcing work values, and articulating high expectations. When employees perceive this supportive and nurturing leadership style, they interpret it as an endorsement from the organization. This boosts their positive emotions, fulfills their need for self-worth, and consequently, elevates their organizational self-esteem. Based on this, the following hypothesis is proposed:

*H2*: Coaching leadership exerts a significant positive influence on organizational self-esteem.

#### 2.1.2 Organizational self-esteem’s influence on employee engagement

Elevated levels of organizational self-esteem positively influence feelings of competence and accomplishment among employees, subsequently enhancing their engagement at work (McAllister and Bigley, 2002). Existing research suggests a strong correlation between organizational self-esteem and work-related attitudes and behaviors (Mauno et al., 2007). Employees with high organizational self-esteem are likely to exhibit greater self-efficacy, increased absorption, and a more optimistic outlook. They are less prone to resource depletion and demonstrate higher levels of engagement (Pierce and Gardner, 2004; Filosa and Alessandri, 2024). On the flip side, those with low organizational self-esteem, often stemming from a lack of organizational support or acknowledgment, are less proactive and show diminished work engagement (Rich et al., 2010). Thus, this paper posits the following hypotheses:

*H3*: Organizational self-esteem significantly positively impacts employee engagement.

*H3a*: Organizational self-esteem positively influences employee vigor.

*H3b*: Organizational self-esteem enhances employee dedication.

*H3c*: Organizational self-esteem contributes to greater employee absorption.

#### 2.1.3 Organizational self-esteem’s mediating role

Drawing from the social exchange theory, employees who are nurtured and valued by their leaders often internalize this appreciation, perceiving themselves as pivotal and influential within the organizational fabric (Pierce et al., 1989). In this reciprocal dynamic, individuals reciprocate the goodwill, reflecting it back to the organization through positive work attitudes. High organizational self-esteem nurtures an optimistic self-image among individuals. Such employees approach setbacks and challenges with optimism, exhibit a heightened sense of duty, and are more inclined to adopt pro-organizational attitudes or behaviors, all to uphold this favorable self-view (McAllister and Bigley, 2002; Jaouadi, 2023), as advocated by Kim et al. (2018) that the corroborates the predictive power of organizational self-esteem on setting organizational expectations. Conversely, low organizational self-esteem often culminates in a pessimistic self-view, which can manifest in counterproductive work behaviors detrimental to the organization (Pierce et al., 1989). Thus, the hypotheses proposed are:

*H4*: Organizational self-esteem acts as a bridge between coaching leadership and employee engagement.

*H4a*: Organizational self-esteem mediates the impact of coaching leadership on employee vigor.

*H4b*: Organizational self-esteem mediates the relationship between coaching leadership and employee engagement.

*H4c*: Organizational self-esteem mediates the nexus between coaching leadership and employee absorption.

#### 2.1.4 The moderating role of learning goal orientation

In the intricate landscape of employee diversity, which encompasses a spectrum of personality traits, needs, and values, the effectiveness of supportive organizational structures in enhancing an individual’s value perception and responsiveness cannot be standardized (Chughtai and Buckley, 2011). Rather, it varies according to individual idiosyncrasies, lending credence to the notion that personal attributes play a significant role in interpreting supportive gestures from leadership (Dweck, 1986). In this context, learning goal orientation emerges as a particularly compelling variable. Distinguished from performance orientation, learning goal orientation emphasizes a continuous pursuit of learning, skill development, and personal growth. Employees with high learning goal orientation inherently believe that their performance is a direct consequence of their efforts (Chughtai and Buckley, 2011) and, when faced with challenges, are more likely to respond proactively, seeking to enhance their skills (Cianci et al., 2010). This outlook engenders a resilient commitment to learning, fostering a culture that values intellectual flexibility and reframes failures as opportunities for future growth (Song et al., 2015). When the goals of leadership align with the goals of such intrinsically motivated employees, the efficacy of a coaching leadership style is markedly increased (Che-Ha et al., 2014). In these synergistic scenarios, employees interpret the supportive gestures and encouragement from leadership as deeply resonant, thereby elevating their organizational self-esteem (Godshalk and Sosik, 2003).

Conversely, employees with low learning goal orientation exhibit a different dynamic (Wang and Hall, 2023). Typically risk-averse and resistant to challenges, these individuals often possess a defeatist attitude when encountering obstacles (Huang and Luthans, 2015). As a result, their intrinsic motivation and enthusiasm for work are limited, even when they are the recipients of robust coaching leadership (Mun and Hwang, 2003). This dampens the potential for a positive impact on their organizational self-esteem (Pierce and Gardner, 2004). However, for individuals propelled by a high learning goal orientation, aligning with coaching leadership surpasses merely enhancing their job satisfaction (Runhaar et al., 2010). Their outstanding performance, fueled by an intrinsic desire for autonomous learning, not only amplifies the effectiveness of coaching leadership but also positions them to receive heightened organizational support, thereby further bolstering their organizational self-esteem. Given these nuanced dynamics, we propose the following hypotheses:

*H5*: Learning goal orientation serves as a moderating factor in the relationship between coaching leadership and organizational self-esteem.

### 2.2 Proposed research model

Based on the theoretical discussions and hypotheses formulated, we outline the subsequent conceptual model to guide our research. The proposed model elucidates the intricate dynamics between coaching leadership, organizational self-esteem, employee engagement, and the modulating effect of learning goal orientation.

## 3 Research design

### 3.1 Variable measurement

To achieve robustness in the study, a multi-faceted approach to variable measurement was adopted. Prior to administering the survey, the respondents were explicitly informed that their responses have no right or wrong answers and would be used exclusively for research purposes. This promoted candid, unbiased responses. A Likert 5-point scoring scale was employed, where “1” represents “strongly disagree,” and “5” represents “strongly agree.” Coaching Leadership (CL) is measured through an 8-item scale developed by Ellinger et al. (2003). Sample item includes: “My supervisor provides me with resources to enhance my job performance effectively.” Organizational self-esteem was assessed using a 10-item scale from Pierce et al. (1989). Sample items include: “In my workplace, I have a great deal of influence.” Employee engagement was evaluated using a 17-item three-dimensional scale by Schaufeli et al. (2002). The scale breaks down into vigor (EE1–EE6), dedication (EE7–EE11), and absorption (EE12–EE17). Sample items include: “I feel energetic while working” and “Once I start working, I become completely absorbed in it.” Learning goal orientation was assessed via a 5-item scale crafted by Vandewalle (1997). Sample items include: “I often look for opportunities to develop new skills and acquire new knowledge.” For specific questions, see Table 1.

**Table 1:** Measurement scale for the impact of coach leadership on employee engagement.

Variable	Code	Measurement item	References source
Coaching leadership	CL1	My leader uses analogies, scenarios, and examples to help me learn.	Ellinger et al. (2003)
	CL2	My leader encourages me to broaden my perspective and understand the bigger picture.	
	CL3	My leader provides constructive feedback to me.	
	CL4	My leader seeks feedback from me to ensure their interactions are helpful to me.	
	CL5	My leader provides resources to help me work more effectively.	
	CL6	My leader helps me think through issues by asking questions rather than giving solutions directly.	
	CL7	My leader sets expectations and communicates the importance of these goals in achieving organizational objectives with me.	
	CL8	My leader helps me see issues from different perspectives by role-playing with me.	
Organizational self-esteem	OSE1	I am influential in the workplace.	Pierce et al. (1989)
	OSE2	I am valued in the workplace.	
	OSE3	I consider myself important in the workplace.	
	OSE4	I am trusted in the workplace.	
	OSE5	People around me have confidence in me at work.	
	OSE6	I am unique in the workplace.	
	OSE7	I am valuable in the workplace.	
	OSE8	I am useful in the workplace.	
	OSE9	I am highly efficient at work.	
	OSE10	I am a good collaborator at work.	
Employee engagement	EE1	When I wake up in the morning, I am eager to go to work.	Schaufeli et al. (2002)
	EE2	I am very energetic when working.	
	EE3	At work, I persevere even when things do not go smoothly.	
	EE4	I can work continuously for a long time.	
	EE5	I feel joyous while working.	
	EE6	I feel energetic at work.	
	EE7	My work is challenging to me.	
	EE8	My work inspires me.	
	EE9	I am passionate about my work.	
	EE10	I take pride in my work.	
	EE11	I find my work very meaningful.	
	EE12	When working, I forget everything around me.	
	EE13	Time flies when I work.	
	EE14	Once I start working, I become fully immersed.	
	EE15	It’s difficult for me to put down my work while working.	
	EE16	I am completely absorbed in my work.	
	EE17	When I am fully engaged in my work, I feel happy.	
Learning goal orientation	LGO1	I am willing to choose challenging tasks and learn a lot from them.	VandeWalle (1997)
	LGO2	I often look for opportunities to expand new skills and acquire new knowledge.	
	LGO3	I enjoy challenging and difficult tasks because they help me learn new skills.	
	LGO4	Developing job skills is important to me, and I am willing to take risks for it.	
	LGO5	I prefer jobs that require high abilities and talents.	

### 3.2 Data collection

The research leveraged the expansive network of MBA and EMBA students at the School of Business, Guizhou University, as a fertile ground for data collection. Utilizing snowball sampling, a total of 494 questionnaires were initially collected. Post the removal of invalid samples, 402 valid questionnaires remained, translating to an effective response rate of 81.38%. The effective sample boasted balanced gender distribution, with females accounting for 53.7% and males making up 46.3%. The most represented age group was 26–30 years (27.4%), and education levels were predominantly bachelor’s degrees (44%). A noteworthy segment of the sample comprised employees from private enterprises (183 participants). Work experience clustered mainly around two intervals: 1–3 years (25.6%) and 4–6 years (37.3%). The respondents’ roles were primarily either ordinary staff (60.7%) or grassroots managers (21.6%). Overall, the surveyed individuals demonstrated the capability to effectively complete the survey questionnaire, thereby ensuring the validity of data collection. Specific descriptive statistics are presented in Table 2.

**Table 2:** Descriptive statistics (*N* = 402).

Demographic features		Frequency	Percentage (%)
Gender	Male	186	46.3
	Female	216	53.7
Age	≤25 years	75	18.7
	26–30 years	110	27.4
	31–35 years	73	18.2
	36–40 years	63	15.7
	41–50 years	42	10.4
	≥50 years	39	9.7
Education level	High school or below	70	17.4
	College diploma	81	20.1
	Bachelor’s degree	177	44.0
	Master’s degree or higher	74	18.4
Company type	State-owned enterprise	81	20.1
	Government agency/public institution	72	17.9
	Private enterprise	183	45.5
	Foreign-funded/joint-venture enterprise	52	12.9
	Other	14	3.5
Years of work	≤1 year	70	17.4
	1–3 years	103	25.6
	4–6 years	150	37.3
	7–10 years	56	13.9
	≥10 years	23	5.7
Position	Ordinary staff	244	60.7
	First-line managers	87	21.6
	Middle managers	55	13.7
	Senior managers	16	4.0

## 4 Research results

### 4.1 Reliability test

Prior to conducting formal regression analysis, it was crucial to establish the reliability of the research data. Utilizing SPSS software, we evaluated the internal consistency of the scales used in this study. As delineated in Table 3, the overall Cronbach’s alpha coefficients for the scales and their respective dimensions all surpassed the 0.7 threshold, signifying acceptable reliability. Additionally, the corrected item-total correlations for each item in the questionnaire exceeded the benchmark of 0.5. Moreover, after the hypothetical removal of individual items, the Cronbach’s alpha coefficients remained lower than the overall Cronbach’s alpha. These findings collectively underscore the high level of reliability of our questionnaire.

**Table 3:** Reliability analysis of the scales.

Variable	Coding	Number of items	Cronbach’s *α* coefficient
Coaching leadership	[CL]	8	0.917
Organizational self-esteem	[OSE]	10	0.935
Employee engagement	[EE]	17	0.939
Vigor	[EE1-6]	6	0.869
Dedication	[EE7-11]	5	0.890
Absorption	[EE12-17]	6	0.901
Learning goal orientation	[LGO]	5	0.859

### 4.2 Validity test

Confirmatory factor analysis was performed to assess the structural validity of the scales. Table 4 presents the results, which include key model fit indices. These indices—*χ*^2^/df (1.388), NFI (0.905), TLI (0.969), and IFI (0.972)—all meet or exceed their respective criteria. The RMSEA value of 0.031 is notably lower than the highest acceptable threshold of 0.08, indicating that the model is a good fit.

**Table 4:** Overall fit indices of the scale.

Statistical test	Measurement value	Standard	Fit result
*χ*^2^/df	1.388	<3	Qualified
NFI	0.905	>0.9	Qualified
TLI	0.969	>0.9	Qualified
IFI	0.972	>0.9	Qualified
RMSEA	0.031	<0.08	Qualified

For evaluating the convergent validity of the questionnaire, we examined the factor loadings, Average Variance Extracted (AVE), and Composite Reliability (CR) for each variable. As Table 5 reveals, the factor loadings for all items corresponding to each variable exceeded the 0.6 mark. This result attests to the high level of representativeness of our overall measurement scale. Furthermore, the AVE values for all variables exceeded the 0.5 threshold, and the CR values were all greater than 0.8. These measurements collectively indicate that the scale has met the standards for convergent validity. Taken together, the analysis of these indicators suggests that the scale has convergent validity that meets the testing standards.

**Table 5:** Convergent validity analysis of the scale.

Path	Estimate	AVE	CR
CL1 ← Coaching leadership	0.734	0.585	0.919
CL2 ← Coaching leadership	0.800		
CL3 ← Coaching leadership	0.795		
CL4 ← Coaching leadership	0.735		
CL5 ← Coaching leadership	0.752		
CL6 ← Coaching leadership	0.719		
CL7 ← Coaching leadership	0.808		
CL8 ← Coaching leadership	0.772		
OSE1 ← Organizational self-esteem	0.796	0.594	0.936
OSE2 ← Organizational self-esteem	0.799		
OSE3 ← Organizational self-esteem	0.746		
OSE4 ← Organizational self-esteem	0.772		
OSE5 ← Organizational self-esteem	0.797		
OSE6 ← Organizational self-esteem	0.761		
OSE7 ← Organizational self-esteem	0.841		
OSE8 ← Organizational self-esteem	0.742		
OSE9 ← Organizational self-esteem	0.739		
OSE10 ← Organizational self-esteem	0.705		
LGO1 ← Learning goal orientation	0.741	0.551	0.860
LGO2 ← Learning goal orientation	0.760		
LGO3 ← Learning goal orientation	0.763		
LGO4 ← Learning goal orientation	0.698		
LGO5 ← Learning goal orientation	0.746		
EE1 ← Vigor	0.751	0.527	0.870
EE2 ← Vigor	0.769		
EE3 ← Vigor	0.651		
EE4 ← Vigor	0.672		
EE5 ← Vigor	0.763		
EE6 ← Vigor	0.742		
EE7 ← Dedication	0.734	0.619	0.890
EE8 ← Dedication	0.839		
EE9 ← Dedication	0.805		
EE10 ← Dedication	0.782		
EE11 ← Dedication	0.770		
EE12 ← Absorption	0.768	0.605	0.902
EE13 ← Absorption	0.770		
EE14 ← Absorption	0.809		
EE15 ← Absorption	0.760		
EE16 ← Absorption	0.789		
EE17 ← Absorption	0.769		

### 4.3 Regression analysis

#### 4.3.1 Hypothesis testing for the influence of coaching leadership on employee engagement

To examine the relationship between coaching leadership and employee engagement, we employed SPSS 25.0 for regression analysis. The detailed results are captured in Table 6. Initially, a baseline model M1-0 was established, featuring employee engagement as the dependent variable and incorporating control variables. Subsequently, we expanded this model into Model M1-1 by introducing coaching leadership as the independent variable. Upon scrutiny of Model M1-1, we found that the coefficient for coaching leadership was both positive and significant (0.506). This demonstrates that, even after accounting for control variables, coaching leadership exerts a substantial positive effect on employee engagement, thereby confirming our first hypothesis (H1). We extended this analytical framework by examining the individual dimensions of employee engagement—namely, Vigor, dedication, and absorption—as separate dependent variables. This approach led to the development of six additional models: M2-0, M2-1 for Vigor; M3-0, M3-1 for dedication; and M4-0, M4-1 for absorption. The results indicate that coaching leadership significantly enhances all three dimensions of employee engagement. Specifically, the coefficients were 0.407 for Vigor, 0.493 for dedication, and 0.438 for absorption. Importantly, introducing coaching leadership as an independent variable in each of these models resulted in a higher change in *R*-squared ∆*R*^2^ compared to the baseline models that included only control variables. This increase in ∆*R*^2^ underscores the model’s enhanced explanatory power, thereby corroborating sub-hypotheses H1a, H1b, and H1c.

**Table 6:** Regression analysis of coaching leadership on employee engagement.

Dependent variable		Employee engagement		Vigor		Dedication		Absorption	
		M1-0	M1-1	M2-0	M2-1	M3-0	M3-1	M4-0	M4-1
Control variables	Gender	−0.034	−0.028	−0.030	−0.025	−0.043	−0.037	−0.018	−0.012
	Age	0.094	0.036	0.114	0.067	0.042	−0.014	0.090	0.040
	Education level	0.047	0.076	0.089	0.112*	0.010	0.038	0.026	0.051
	Company type	0.084	0.057	0.114*	0.092*	0.084	0.057	0.028	0.004
	Years of work	0.035	0.036	0.007	0.008	0.034	0.035	0.049	0.050
	Position	0.199***	0.119**	0.204***	0.139**	0.154**	0.077	0.167**	0.098*
Independent variable	CL		0.506***		0.407***		0.493***		0.438***
	*R*^2^	0.077	0.317	0.081	0.236	0.049	0.276	0.057	0.237
	∆*R*^2^	0.063	0.305	0.067	0.222	0.034	0.263	0.043	0.223
	*F*	5.510***	26.118***	5.792***	17.381***	3.382**	21.428***	4.007**	17.478***

*p < 0.05, **p < 0.01, ***p < 0.001.

#### 4.3.2 Initial examination of the impact of coaching leadership on organizational self-esteem

To delve into the mediating effect of organizational self-esteem in the relationship between coaching leadership and employee engagement, we conducted a series of regression analyses using SPSS 25.0. The pertinent outcomes are elaborated in Tables 7, 8. Initially, we constructed a base model, designated as Model M5-0, where organizational self-esteem served as the dependent variable and control variables were included. Building upon this, we introduced coaching leadership as the independent variable, resulting in Model M5-1. Evaluation of Model M5-1 revealed that the coefficient associated with coaching leadership was positively significant at 0.523. This substantiates that coaching leadership exerts a strong positive influence on organizational self-esteem after accounting for the control variables. Hence, Hypothesis H2 receives empirical support.

**Table 7:** Regression analysis of coaching leadership on organizational self-esteem.

Dependent variable		Organizational self-esteem	
		M5-0	M5-1
Control variables	Gender	−0.064	−0.058
	Age	0.100	0.040
	Education level	−0.031	−0.001
	Company type	0.068	0.040
	Years of work	0.015	0.016
	Position	0.195***	0.113*
Independent variable	CL		0.523***
	*R*^2^	0.086	0.342
	∆*R*^2^	0.072	0.331
	*F*	6.207***	29.295***

*p < 0.05, **p < 0.01, ***p < 0.001.

**Table 8:** Regression analysis of coaching leadership, organizational self-esteem, and employee engagement.

Dependent variable		Employee engagement	
		M5-2	M5-3
Control variables	Gender	−0.034	−0.008
	Age	0.094	0.022
	Education level	0.047	0.076
	Company type	0.084	0.043
	Years of work	0.035	0.030
	Position	0.199***	0.080
Independent variables	CL		0.325***
Mediating variable	OSE		0.347***
	*R*^2^	0.077	0.396
	∆*R*^2^	0.063	0.384
	*F*	5.510***	32.245***

*p < 0.05, **p < 0.01, ***p < 0.001.

#### 4.3.3 Analysis of the mediated relationship through organizational self-esteem

In a subsequent analysis aimed at unpacking the mediated effects, we used employee engagement as the dependent variable. A baseline model, named Model M5-2, was crafted, incorporating control variables. Extending from this, Model M5-3 was formulated by including both the independent variable, coaching leadership, and the mediating variable, organizational self-esteem. Close examination of Model M5-3 revealed that the coefficients for both coaching leadership and organizational self-esteem were positive and significant. Intriguingly, the inclusion of organizational self-esteem as a mediator led to a decline in the primary effect of coaching leadership on employee engagement, from a coefficient of 0.506 down to 0.325. This suggests that organizational self-esteem not only positively influences employee engagement but also partially mediates the effect of coaching leadership on engagement. As a result, Hypotheses H3 and H4 are corroborated.

#### 4.3.4 Further validation of mediating effect through bootstrap analysis

To bolster the validity of our findings, this study utilizes the Process v4.0 plugin and employed a Bootstrap method with a 95% confidence interval and 5,000 bootstrap samples. According to established statistical norms, an effect is deemed significant if the confidence interval does not include zero. The detailed empirical outcomes are presented in Table 9. The Bootstrap analysis revealed that the total effect of coaching leadership on employee engagement is significant, with a coefficient *β* = 0.4487 and a 95% confidence interval [0.3736, 0.5237] that does not include zero. Importantly, even upon the introduction of organizational self-esteem as a mediating variable, the direct effect of coaching leadership on employee engagement remains significant *β* = 0.2876, 95% CI = [0.2044, 0.3708]. Additionally, the analysis confirms a significant indirect effect of organizational self-esteem in the relationship between coaching leadership and employee engagement *β* = 0.1611, 95%CI = [0.0953, 0.2395]. This lends further credence to Hypotheses H3 and H4, affirming that organizational self-esteem functions as a partial mediator between coaching leadership and employee engagement.

**Table 9:** Bootstrap test of the mediation effect of organizational self-esteem.

	Path	Effect value	Standard error	Lower bound	Upper bound
Direct effect	CL → EE	0.2876	0.0423	0.2044	0.3708
Indirect effect	CL → OSE → EE	0.1611	0.0365	0.0953	0.2395
Total effect	CL → EE	0.4487	0.0382	0.3736	0.5237

#### 4.3.5 Examination of the mediating effect on vigor

Continuing our investigation, a separate regression analysis was conducted to study the influence of coaching leadership and organizational self-esteem on Vigor. The findings are summarized in Table 10. Initially, we formulated Model M6-0 with Vigor as the dependent variable and added control variables. This model was subsequently expanded to include both the independent variable of coaching leadership and the mediating variable of organizational self-esteem, resulting in Model M6-1. Upon scrutinizing Model M6-1, we found that both coaching leadership and organizational self-esteem had positive and significant coefficients. The analysis thus demonstrates that organizational self-esteem positively predicts Vigor and serves as a partial mediator between coaching leadership and Vigor, substantiating Hypotheses H3a and H4a.

**Table 10:** Regression analysis of coaching leadership and organizational self-esteem on vigor.

Dependent variable		Vigor	
		M6-0	M6-1
Control variables	Gender	−0.030	−0.004
	Age	0.114	0.053
	Education level	0.089	0.112*
	Company type	0.114*	0.077
	Years of work	0.007	0.002
	Position	0.204***	0.098*
Independent variables	CL		0.216***
Mediating variable	OSE		0.365***
	*R*^2^	0.081	0.323
	∆*R*^2^	0.067	0.310
	*F*	5.792***	23.485***

*p < 0.05, **p < 0.01, ***p < 0.001.

As detailed in Table 11, the total effect of coaching leadership on vigor is significant *β* = 0.3822, 95%CI = [0.2982, 0.4662]. Furthermore, the direct effect of coaching leadership on vigor remains robust even after including the mediating variable *β* = 0.2030, 95%CI = [0.1097, 0.2964]. Finally, the indirect effect of organizational self-esteem between coaching leadership and vigor is also significant *β* = 0.1792, 95%CI = [0.1061, 0.2627]. In sum, the Bootstrap analysis and the further regression models confirm that organizational self-esteem acts as a partial mediator in the relationships between coaching leadership and both employee engagement and vigor. This corroborates Hypotheses H3, H4, H3a, and H4a.

**Table 11:** Bootstrap test of organizational self-esteem mediation effect (part one).

	Path	Effect value	Standard error	Lower bound	Upper bound
Direct effect	CL → Vigor	0.2030	0.0475	0.1097	0.2964
Indirect effect	CL → OSE → Vigor	0.1792	0.0403	0.1061	0.2627
Total effect	CL → Vigor	0.3822	0.0427	0.2982	0.4662

#### 4.3.6 Exploration of the mediating effect on dedication

In line with the analytical steps used in previous models, we examined dedication as a dependent variable. Initially, control variables were added to create Model M7-0. Subsequently, Model M7-1 was formulated by including both the independent variable—coaching leadership—and the mediating variable—organizational self-esteem. These results are elaborated in Table 12. Upon analyzing Model M7-1, we observed that both coaching leadership and organizational self-esteem had positive and significant coefficients. This leads to the conclusion that organizational self-esteem has a positive influence on employee dedication and serves as a partial mediator between coaching leadership and dedication. This confirms Hypotheses H3b and H4b.

**Table 12:** Regression analysis of coaching leadership and organizational self-esteem on dedication.

Dependent variable		Dedication	
		M7-0	M7-1
Control variables	Gender	−0.043	−0.022
	Age	0.042	−0.025
	Education level	0.010	0.038
	Company type	0.084	0.047
	Years of work	0.034	0.031
	Position	0.154**	0.046
Independent variables	CL		0.352***
Mediating variable	OSE		0.269***
	*R*^2^	0.049	0.323
	∆*R*^2^	0.034	0.310
	*F*	3.382**	23.485***

*p < 0.05, **p < 0.01, ***p < 0.001.

As detailed in Table 13, the total effect of coaching leadership on dedication is significant *β* = 0.5319, 95%CI = [0.4378, 0.6260]. Furthermore, even after accounting for the mediating effect of organizational self-esteem, the direct influence of coaching leadership on dedication remains significant *β* = 0.3797, 95%CI = [0.2723, 0.4870]. Moreover, organizational self-esteem shows a significant indirect effect between coaching leadership and dedication *β* = 0.1522, 95%CI = [0.0721, 0.2453]. In conclusion, organizational self-esteem partially mediates the relationship between coaching leadership and dedication, confirming H3b and H4b.

**Table 13:** Bootstrap test of organizational self-esteem mediation effect (part 2).

	Path	Effect value	Standard error	Lower bound	Upper bound
Direct effect	CL → Dedication	0.3797	0.0546	0.2723	0.4870
Indirect effect	CL → OSE → Dedication	0.1522	0.0436	0.0721	0.2453
Total effect	CL → Dedication	0.5319	0.0479	0.4378	0.6260

#### 4.3.7 Examination of the mediating effect on absorption

Analysis of Model M8-1 in Table 14 indicates that the coefficients for coaching leadership and organizational self-esteem are positive and significant. Consequently, we infer that organizational self-esteem positively affects employee absorption and acts as a partial mediator between coaching leadership and absorption, corroborating Hypotheses H3c and H4c.

**Table 14:** Regression analysis of coaching leadership and organizational self-esteem on absorption.

Dependent variable		Absorption	
		M8-0	M8-1
Control variables	Gender	−0.018	0.004
	Age	0.090	0.029
	Education level	0.026	0.051
	Company type	0.028	−0.007
	Years of work	0.049	0.046
	Position	0.167**	0.066
Independent variables	CL		0.290***
Mediating variable	OSE		0.282***
	*R*^2^	0.057	0.289
	∆*R*^2^	0.043	0.275
	*F*	4.007**	20.007***

*p < 0.05, **p < 0.01, ***p < 0.001.

**Figure 1:**
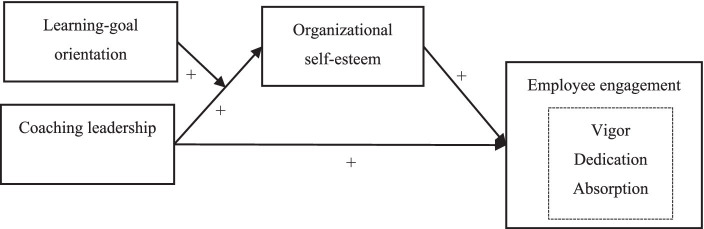
Research model. “+” represents a positive impact.

As reported in Table 15, the total effect of coaching leadership on absorption is noteworthy *β* = 0.4457, 95%CI = [0.3547, 0.5367]. In addition, the direct effect of coaching leadership on absorption remains robust even when the mediating role of organizational self-esteem is considered *β* = 0.2954, 95%CI = [0.1917, 0.3990]. Lastly, organizational self-esteem exerts a significant indirect influence between coaching leadership and absorption *β* = 0.1503, 95%CI = [0.0718, 0.2430]. In conclusion, our further analyses indicate that organizational self-esteem plays a partial mediating role in the relationship between coaching leadership and both dedication and absorption. These results solidify the support for Hypotheses H3b, H4b, H3c, and H4c, expanding the overall robustness of our theoretical framework (Figure 1).

**Table 15:** Bootstrap test of organizational self-esteem mediating effects (part three).

	Path	Effect value	Standard error	Lower bound	Upper bound
Direct effect	CL → Absorption	0.2954	0.0527	0.1917	0.3990
Indirect effect	CL → OSE → Absorption	0.1503	0.0436	0.0718	0.2430
Total effect	CL → Absorption	0.4457	0.0463	0.3547	0.5367

#### 4.3.8 Testing the moderating effect of learning goal orientation

To explore the moderating effects of learning goal orientation on the relationship between coaching leadership and organizational self-esteem, we employed linear regression with a product term approach. Prior to the analysis, the data were centered to facilitate the interpretation of interaction terms. The analysis aimed to test hypothesis H5 and the specific outcomes are presented in Table 16. After accounting for control variables, the main effects of coaching leadership and learning goal orientation, as well as their interaction term, were included in the model. Notably, the coefficient for the interaction term was positive and significant *β* = 0.108. This result suggests that learning goal orientation amplifies the positive influence of coaching leadership on organizational self-esteem, thereby confirming hypothesis H5.

**Table 16:** Regression analysis of learning goal orientation moderating effects on organizational self-esteem.

Dependent variable	Organizational self-esteem		
	M9-0	M9-1	M9-2
Gender	−0.064	−0.063	−0.066
Age	0.100	0.049	0.035
Education level	−0.031	0.001	0.003
Company type	0.068	0.023	0.026
Years of work	0.015	0.014	0.022
Position	0.195***	0.083	0.082
CL		0.420***	0.447***
LGO		0.269***	0.291***
CL*LGO			0.108*
*R*^2^	0.086	0.401	0.411
∆*R*^2^	0.072	0.389	0.398
*F*	6.207***	32.944***	30.422***

*p < 0.05, **p < 0.01, ***p < 0.001.

**Figure 2:**
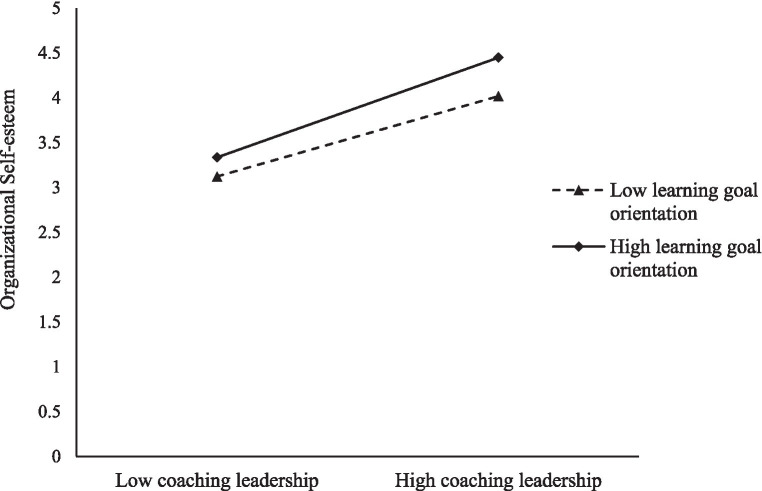
Moderating effect of learning goal orientation between coaching leadership and organizational self-esteem.

To elucidate the moderation effect visually, this study employed Model 7 from SPSS Process v4.0 and executed 5,000 bootstrapped resamples for more robust results. The analysis was conducted within a 95% confidence interval. Figure 2 graphically presents the moderation effects at different levels of learning goal orientation. The analysis reveals two critical points: When learning goal orientation is low, the regression slope connecting coaching leadership and organizational self-esteem is relatively gentle, indicating a subdued positive influence. In contrast, at high levels of learning goal orientation, the regression slope becomes considerably steeper. This suggests a stronger, more substantial positive effect of coaching leadership on organizational self-esteem. In summary, learning goal orientation serves as a significant moderator that enhances the positive relationship between coaching leadership and organizational self-esteem. This validates our hypothesis H5 and enriches the study’s overall theoretical model.

## 5 Research conclusion and discussion

### 5.1 Findings

Our study, leveraging a sample of 402 valid responses from MBA and EMBA students at the School of Business, Guizhou University, aimed to dissect the nuanced interplay between coaching leadership, employee engagement, organizational self-esteem, and learning goal orientation. The findings largely substantiate our proposed hypotheses, illuminating several complex mechanisms that underlie the effectiveness of coaching leadership in fostering employee engagement.

#### 5.1.1 Coaching leadership’s positive impact on employee engagement

Firstly, in alignment with Hypotheses H1–H1c, we found compelling evidence through linear regression analysis that coaching leadership has a direct and significant positive impact on employee engagement. Particularly in today’s VUCA (Volatile, Uncertain, Complex, Ambiguous) landscape, coaching leadership stands out as a transformative approach. This leadership style focuses less on providing direct solutions (“giving fish”) and more on empowering employees to problem-solve independently (“teaching how to fish”). Our data affirm that such an approach better engages employees’ intrinsic motivations, consequently enhancing their dedication and absorption in the workplace, which is precisely the synergistic effect of coach-based leadership on employee engagement highlighted in this research. Therefore, organizational leaders should prioritize caring for and nurturing employees, mastering team communication skills. They should increasingly employ encouraging, guiding, and inspirational communication methods to stimulate employees’ intrinsic work motivation, thereby achieving mutual development between the organization and its employees.

#### 5.1.2 Mediating role of organizational self-esteem

Secondly, our analysis supports Hypotheses H2–H4, which posited that organizational self-esteem serves as a pivotal mediating variable in this dynamic. We found that coaching leadership goes beyond merely influencing work-related behaviors; it also has a psychological ripple effect. Specifically, the coaching leadership style—which centers on positive communication, emotional support, and professional development—leads to heightened organizational self-esteem among employees. Elevated levels of organizational self-esteem subsequently translate into a more optimistic work attitude and increased dedication, further underscoring the multi-layered influence of coaching leadership. Furthermore, coaching leadership fundamentally emphasizes encouragement, support, positive communication interactions, and a focus on employees’ career development. This leadership approach effectively improves employees’ self-awareness, enhances their positive self-concept, and ultimately rewards the organization through dedication and focused work contributions.

#### 5.1.3 Moderating effect of learning goal orientation

Lastly, as elucidated by Hypothesis H5, learning goal orientation functions as a key moderating variable. Our data suggest that the benefits of coaching leadership are most pronounced among employees with a high learning goal orientation. Compared with lower-level goal-oriented individuals, higher-level goal-oriented individuals spontaneously have a higher level of learning and improvement motivation. With the support of coaching leadership style, individuals will be aroused more positive goal orientation and strong self-drive. Therefore, the support and encouragement factors of coach leadership can induce the resonance of high-level learning goal-oriented individuals. Their strong affinity for this leadership style not only boosts their perception of organizational support but also bolsters their engagement and job performance. However, it is worth noting that those with low learning goal orientation might not be as responsive to the empowering elements of coaching leadership, revealing an area that warrants further managerial attention.

### 5.2 Management recommendations

#### 5.2.1 Operationalizing coaching leadership

Organizations keen to cultivate a coaching leadership style should focus on three critical human resource pillars: recruitment, performance assessment, and continuous training. During recruitment, candidates should be assessed for their alignment with coaching leadership traits, employing psychometric tools, situational simulations, and in-depth interviews. Post-hiring, performance metrics need to be redefined to incentivize coaching-oriented behaviors. Assessment criteria should encapsulate leaders’ effectiveness in nurturing employees’ psychological potential and promoting positive workplace interactions. On the training front, a blend of experiential activities like role-playing can be used to refine existing managerial styles toward a coaching paradigm.

#### 5.2.2 Elevating organizational self-esteem

Fostering a culture that bolsters employees’ organizational self-esteem is paramount. Reward mechanisms should recognize and incentivize standout performances, thereby enhancing individual and collective psychological well-being. Additionally, fostering teamwork and facilitating frequent knowledge exchange can provide employees the platform to feel integral to the organization’s fabric, thereby enhancing their willingness to take on challenging tasks and exhibit innovative behaviors.

#### 5.2.3 Strategizing around learning goal orientation

The utility of learning goal orientation in talent management cannot be overstated. During recruitment, personality tests can identify individuals with a predisposition toward a high learning goal orientation. Subsequent in-house training should be tailored to nurture this quality further. For example, employees with a high learning goal orientation can be assigned complex, challenging tasks that align with their intrinsic motivations, thereby benefiting both the individual and the organization.

### 5.3 Theoretical implications

Our findings contribute to a more comprehensive understanding of how coaching leadership, organizational self-esteem, and learning goal orientation interact to impact employee engagement and dedication. We integrate these diverse theoretical strands, providing a nuanced view of leadership effectiveness, particularly in the VUCA context.

Firstly, our research extends the theoretical framework regarding leadership efficacy, especially in modern complex organizational environments. By exploring the relationship between coaching leadership, organizational self-esteem, learning goal orientation, and employee engagement, we reveal a new leadership paradigm that helps address the constantly changing work environment. This theoretical contribution offers organizational managers a more comprehensive and adaptable leadership approach.

Secondly, our study enriches the theoretical framework concerning coaching leadership and employee engagement. By examining the impact of coaching leadership, organizational self-esteem, and learning goal orientation on employee engagement and loyalty, we gain deeper insights into the mechanisms underlying employee behaviors and attitudes in the workplace. This provides guidance for organizational managers to better motivate and cultivate employees, thus enhancing organizational performance.

Lastly, our research provides insights for addressing leadership challenges in the VUCA environment. In this environment of uncertainty and complexity, effective practices of coaching leadership can assist organizations in building more flexible and adaptive teams. Furthermore, emphasizing the importance of learning goal orientation can encourage employees to maintain a mindset of learning and growth amidst constant change, facilitating better adaptation. Therefore, our study offers practical guidance and theoretical support for organizations in addressing VUCA challenges.

### 5.4 Practical implications

Practically, this study emphasizes the imperative for organizational leaders to embrace coaching leadership styles, considering their discernible influence on employee engagement. It suggests the development of training programs tailored to equip supervisors with the requisite skills. Furthermore, nurturing a culture of high organizational self-esteem and fostering learning goal orientation can serve as force multipliers in augmenting employee engagement. Expanding on this, organizations could implement comprehensive coaching leadership training modules that focus on enhancing communication skills, providing constructive feedback, and facilitating employee development. These programs should be designed to empower leaders to effectively mentor and guide their teams, fostering a supportive work environment conducive to continuous learning and growth. Additionally, organizational initiatives aimed at promoting a positive organizational culture, such as recognition programs and opportunities for skills enhancement, can reinforce the importance of organizational self-esteem and learning goal orientation. By aligning leadership practices with these principles and values, organizations can cultivate a workforce that is not only highly engaged but also motivated to contribute to the organization’s success.

### 5.5 Research limitations and future outlook

The scope of this study is accompanied by specific limitations that merit acknowledgment while also opening avenues for future inquiry. In the context of sample distribution, this research opted for a generalist approach, forgoing an in-depth examination of industry-specific or group-focused dynamics. This choice might dilute the applicability of the findings across varying sectors and demographic clusters. As such, a sectoral focus—zeroing in on domains like education, healthcare, real estate, or the tech industry—could enrich the granularity of future investigations, offering more tailored insights.

Moreover, this study primarily emphasizes the positive variables that modulate the relationship between coaching leadership and employee engagement. While this focus yields valuable insights, there is a conspicuous absence of research concerning negative influences. Future studies should broaden the investigatory lens to encompass not only facilitative variables like learning goal orientation but also inhibitory factors such as abusive leadership, emotional exhaustion, and workplace loneliness. A more multidimensional analytical approach, potentially employing different hierarchical levels of variables, would offer a more nuanced understanding of the mechanisms through which coaching leadership impacts employee engagement.

Lastly, the methodological framework adopted for this research, while robust, could be further refined for enhanced scientific rigor. Future research endeavors could exploit a multifaceted methodological toolkit comprising paired research designs, controlled experiments, and detailed case studies. Such an integrative approach would substantially elevate the validity and reliability of the research outcomes.

### 5.6 Conclusion

Overall, our findings provide valuable insights into the multifaceted impact of coaching leadership on employee engagement, mediated by organizational self-esteem and moderated by learning goal orientation. These results not only further academic dialogue but also provide actionable insights for practitioners, emphasizing the symbiotic relationship between effective leadership and a committed workforce.

## Data availability statement

The raw data supporting the conclusions of this article will be made available by the authors, without undue reservation.

## Ethics statement

Ethical approval was not required for the study involving humans in accordance with the local legislation and institutional requirements. Written informed consent to participate in this study was not required from the participants or the participants’ legal guardians/next of kin in accordance with the national legislation and the institutional requirements.

## Author contributions

LT: Conceptualization, Funding acquisition, Methodology, Writing – original draft. MS: Data curation, Formal analysis, Investigation, Methodology, Writing – original draft. YuL: Conceptualization, Data curation, Methodology, Writing – original draft. YiL: Conceptualization, Data curation, Formal analysis, Methodology, Supervision, Writing – original draft. BY: Conceptualization, Methodology, Supervision, Writing – original draft, Writing – review & editing.
